# Energy metabolism in different skeletal muscles and muscle fibers: implications for injury and dietary supplementation

**DOI:** 10.1007/s00424-025-03112-5

**Published:** 2025-09-27

**Authors:** Andrey V. Kuznetsov, Raimund Margreiter, Judith Hagenbuchner, Michael J. Ausserlechner

**Affiliations:** 1 3D Bioprinting Core Facility, Department of Pediatrics I, Medical University of Innsbruck, Innsbruck, Austria; 2 Department of Visceral, Transplant and Thoracic Surgery, Medical University of Innsbruck, A-6020 Innsbruck, Austria

**Keywords:** Bioenergetics, Cardiac/skeletal muscles energy metabolism, Creatine/phosphocreatine, Intracellular energy transport, Mitochondria, Mitochondrial ROS

## Abstract

The necessary energy supply in skeletal muscles is based on either glycolysis or mitochondrial oxidative phosphorylation (OxPhos). These two bioenergetic pathways are in balanced complementation. Glycolysis is faster than OxPhos, whereas OxPhos is much more efficient. One common feature of both pathways is the compartmentation of high-energy phosphates and their metabolic channeling. The glycolytic muscles are wider, whereas oxidative muscles have significantly more mitochondria. Importantly, a striking difference in bioenergetic mechanisms in oxidative (slow-twitch) versus glycolytic (fast-twitch) muscles and muscle fibers has been clearly shown. The advantage is that the optimal fiber diversity can provide the best muscle function. Various creatine kinase isoforms and phosphocreatine play an important role in glycolytic and oxidative muscles energy metabolism, but their roles are very different, depending on the muscle type. In the glycolytic muscles, phosphocreatine, produced from creatine and ATP by cytosolic creatine kinase, is mostly considered a cellular energy store for fast ATP delivery, whereas in the oxidative muscles, phosphocreatine and mitochondrial creatine kinase are the main players in the intracellular energy transport.

## Introduction

It is known that the cellular levels of ATP in various muscles are rather limited; so they are unable to support the permanent muscle contraction. Therefore, some bioenergetic pathways must be used to continuously supply ATP. This includes glycolysis (anaerobic) and/or mitochondrial OxPhos (aerobic). The energy is required not only for muscle contraction but also for the activity of key cellular enzymes, ion pumping in sarcolemma, endoplasmic reticulum, etc. [24]. Importantly, glycolysis and mitochondrial OxPhos have their own specific features and advantages. Glycolysis is more operative under no-oxygen conditions or in cells without mitochondria. This pathway is 100 times faster than OxPhos. On the other hand, OxPhos is much more efficient (substantially more ATP is produced, using fatty acids as a substrate (~ 20 times more). That means that, in the actions demanding long exercise times (but at submaximal capacities, like in the myocardium, marathon runners’ muscles), energy can be provided mostly by fatty acid-supported mitochondrial OxPhos. This means that oxidative muscles/fibers contain a higher mitochondrial amount and correspondingly more mitochondrial creatine kinase isoform (mitCK). At the same time, the activity of cytosolic CK (MM isoform) is much higher in glycolytic muscles, which is necessary during muscle contraction at high intensity, when ATP is quickly regenerated from the cellular pool of phosphocreatine (PCr). A common feature of both pathways is compartmentation of high-energy phosphates (ATP, PCr) and direct metabolic channeling. This suggests that the mechanisms by which ATP is supplied for muscle contraction require a highly organized cellular system in both glycolytic and OxPhos pathways. These pathways are not only highly regulated, but they are interconnected, and the inhibition of OxPhos activates glycolysis, whereas increased fatty acid and glucose availability to mitochondria during exercise decreases muscle glycogen utilization and glycolysis. The review discusses our understanding of the bioenergetic mechanism features in different muscles and muscle fibers under normal, pathological, and various nutrition conditions.

## The affinity of mitochondria to the main mitochondrial substrate ADP is very different in low-twitch (oxidative) and fast-twitch (glycolytic) skeletal muscles

Importantly, striking differences in the regulation mechanisms of mitochondrial respiration in oxidative and glycolytic muscles in situ have been found using permeabilized saponin muscle fibers [3, 32, 48]. The apparent Michaelis constant (ap-pKm) for ADP is considered an important mitochondrial parameter of cellular energy metabolism and mitochondrial bioenergetics. It shows the affinity of mitochondrial respiration to the main regulator—ADP, and thus revealing the permeability state of the outer mitochondrial membrane. A low appKm(ADP), about 15 to 300 µM (high response to ADP) has been found for isolated mitochondria in many reports [32]. However, measurements of this parameter in permeabilized cells or oxidative muscle fibers (heart, soleus, etc.) showed appKm(ADP) in a range of 200 to 3500 µM (more than tenfold higher value). This was explained by the interactions of mitochondria with some elements of the cytoskeleton, such as tubulin beta II [23], possibly desmin [43] and plectin [49, 63].

Table 1 demonstrates that permeabilized muscle fibers, isolated from different muscle types (oxidative and glycolytic) show a dramatic difference in the sensitivity to the main mitochondrial regulator ADP, expressed as appKm(ADP) (examples, could be soleus, myocardium and gastrocnemius [32]). In addition, it has been shown that there is some difference in the maximal rates of mitochondrial respiration (VO_2_max per mg of wet weight, glutamate/malate have used as mitochondrial substrates) in the permeabilized oxidative and glycolytic muscle fibers, assuming a higher content of mitochondria in oxidative fibers. The low sensitivity to ADP in oxidative muscles can be significantly increased in the presence of creatine (Table 1). This was explained by the functional, and probably also structural, coupling of adenine-nucleotide translocase (ANT) in the inner mitochondrial membrane and mitochondrial creatine kinase (mitCK) in the intermembrane space [49] (for more details see below). This is especially important, considering strong evidence of the enzymatic and metabolic channeling, including enzyme redistributions and substrates/nucleotides (ADP/ATP) cellular micro-compartmentations [29, 52, 55].

**Table 1:** Mitochondrial affinity for ADP (appKm for ADP) and maximal oxygen consumption rates in various types of muscle (see also Kuznetsov [32])

Muscles	appKm (ADP), µM	Vmax, ng-atoms O.min^−1^.mg^−1^
Isolated rat heart mitochondria	15–30	250–300
Permeabilized rat myocardial fibers	300 ± 35	29 ± 1.5
Permeabilized rat myocardial fibers + 20-mM creatine	85 ± 5	28 ± 4.0
Permeabilized muscle soleus fibers	350 ± 50	12.5 ± 0.5
Permeabilized soleus fibers + 20-mM creatine	105 ± 15	15 ± 4
Permeabilized quadriceps fibers	25 ± 5	8 ± 4
Permeabilized gastrocnemius fibers	13 ± 2.6	7.0 ± 1.1

Mitochondrial affinity for ADP (appKm for ADP, µM) was measured from ADP kinetics and maximal oxygen consumption rates (VO2max) was evaluated by O2 high-resolution polarography in various types of muscles in muscle fibers permeabilized by 50 µg/ml of saponin [32, 35, 48].

## Imaging of mitochondria and mitochondrial function in different muscle fibers

### Confocal fluorescent microscopy

Confocal fluorescent microscopy permits analysis of mitochondrial endogenous flavoprotein autofluorescence, mitochondrial NAD(P)H, and specific mitochondrial fluorescent probes in permeabilized muscle fibers, providing precise imaging of mitochondria in single fibers [33].

**Fig. 1:**
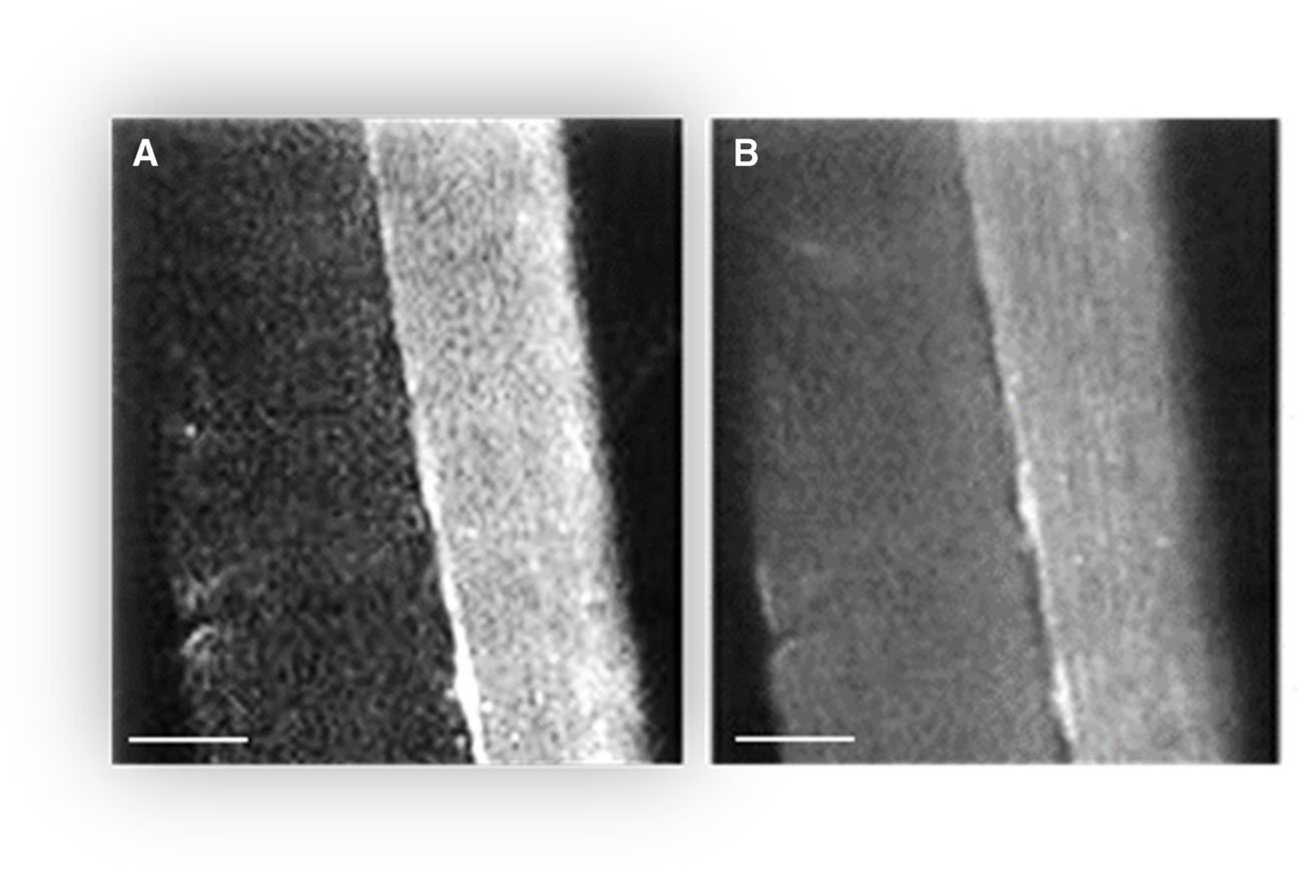
Confocal fluorescent imaging of (**A**) the flavoprotein autofluorescence (488 nm excitation, 530 nm emission) and (**B**) the fluorescence of the mitochondrial-specific dye DASPMI (488 nm excitation, 520 nm emission) in the different saponin-permeabilized mouse quadricep muscle fibers scale bar, 50 µm

**Fig. 2:**
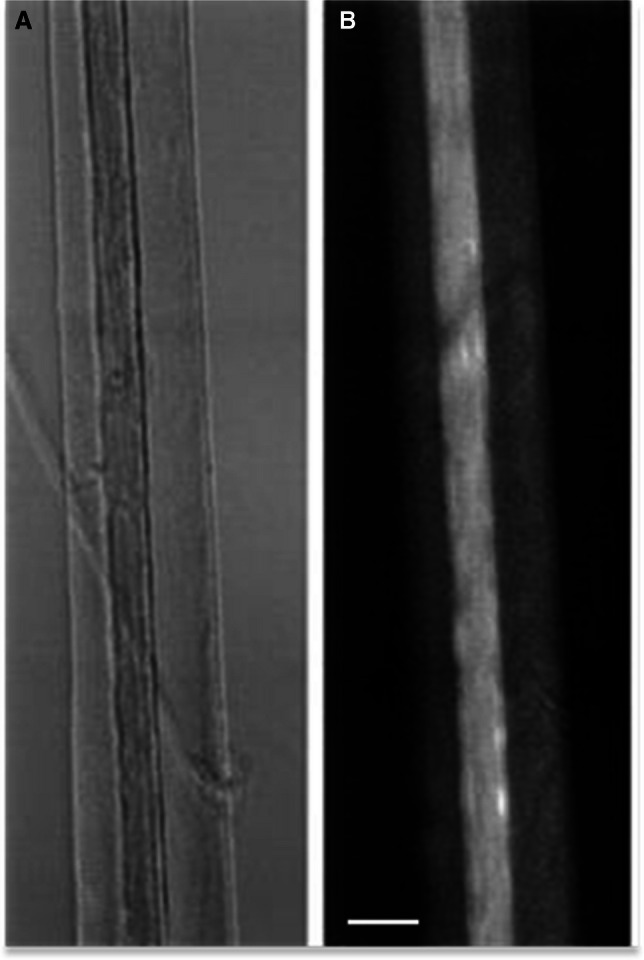
Optical phase-contrast (**A**) and flavoprotein/NADH ratio (**B**) microscopy of the permeabilized mouse quadriceps fibers (NADH; 325 nm excitation); scale bar, 50 µm

Figure 1A and B shows the representative images of the permeabilized mice quadriceps fiber bundle. The intensities of the flavoproteins fluorescence signals (in fully oxidized state) of individual fibers are significantly different and much higher in oxidative fiber (Fig. 1A). Similarly, Fig. 1B demonstrates a much higher signal of the potentiometric mitochondria-specific probe dimethylaminostyryl pyridyl methyl iodide (DASPMI) in this fiber. A single fiber with bright fluorescence presumably represents an oxidative, mitochondria-rich fiber (Fig. 1). Another neighboring fiber (left fiber), that is in the same oxidized state, evidently has a significantly weaker signal and therefore contains less mitochondria. It is noteworthy that the imaging approach enables not only the observation of mitochondrial structure/morphology but also the ability to obtain information about the redox state and the general mitochondrial functions. Since mitochondrial flavoproteins are fluorescent in an oxidized state and NAD(P)H in a reduced state, it has been possible to continuously monitor mitochondrial redox states. Importantly, the flavoprotein signal in skeletal fibers and other cells was always colocalized with a specific and widely used marker of mitochondria MitoTracker™ Green FM [33, 44, 57]. Moreover, a different size (diameter) of these fibers can also be seen (Fig. 1). Glycolytic fibers are wider (in diameter) and more adaptive to exercise (e.g., glycolytic gastrocnemius muscle fibers are wider than oxidative soleus muscle fibers [34, 35]). Figure 2B shows a much more sensitive flavoprotein/NADH ratio confocal microscopy of the permeabilized mouse quadriceps fibers. It can be seen that there are two glycolytic and one oxidative fiber (in the middle). Importantly, this ratio does not depend on any other fluorescence signals.

## The role of phosphocreatine for the efficient intracellular energy storage and transfer in different skeletal muscles

**Fig. 3:**
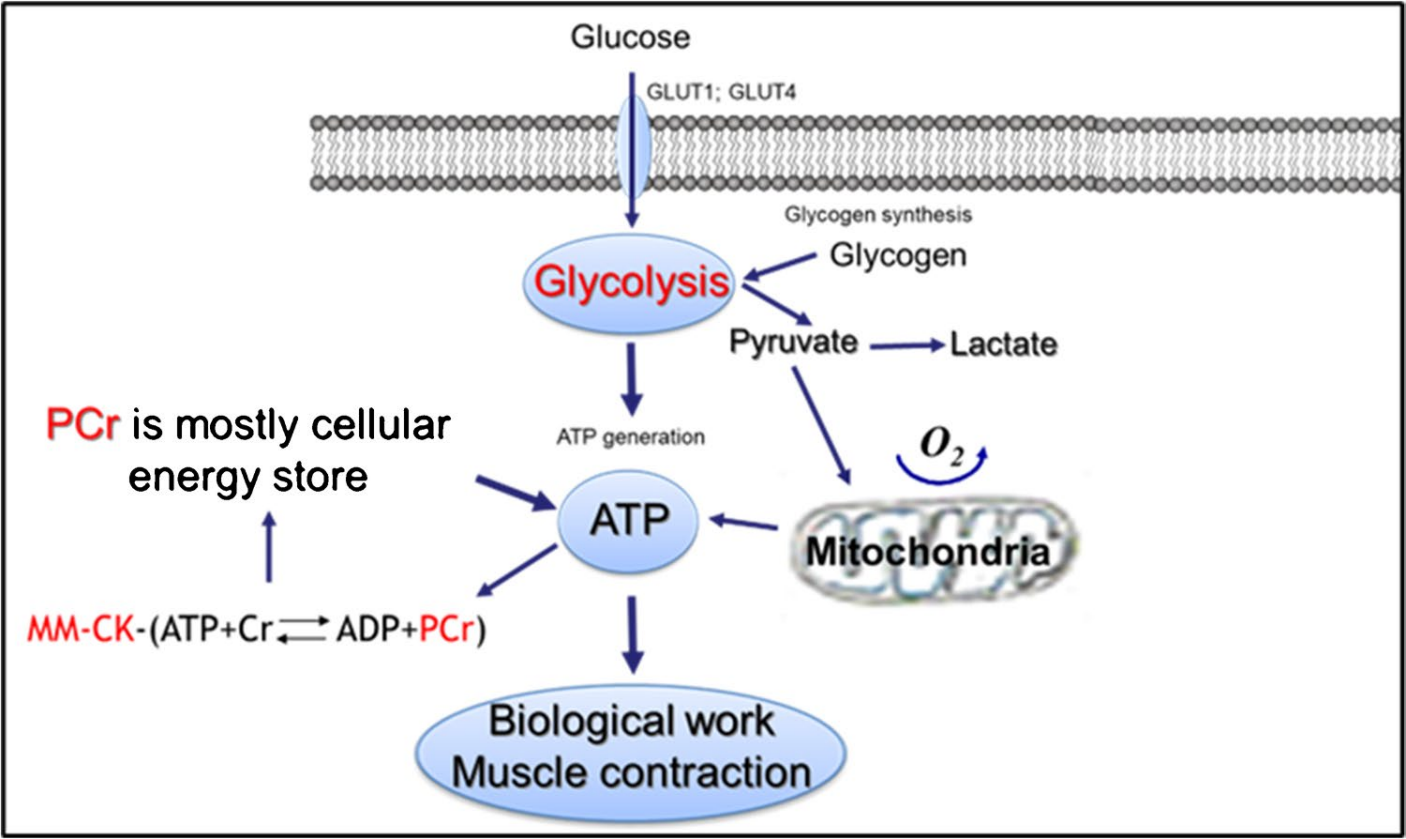
Energy metabolism mechanisms in glycolytic, fast-twitch skeletal muscles

The important aspect of muscle energy metabolism, in various muscles, is the capacity for rapid recovery of high intracellular phosphocreatine (PCr) levels, if it drops to low (minimal) levels. In glycolytic muscles, this recovery occurs from the glycolytic or OxPhos-generated ATP and cytosolic creatine kinase (CK) reaction (Cr + ATP > PCr + ADP). Fast (in seconds) ATP re-synthesis is needed for their contraction and is produced from the large pool of cytosolic PCr and high activity of the muscular isoform of creatine kinase (MMCK), from the reversed reaction PCr + ADP > Cr + ATP. Therefore, in glycolytic, fast-twitch muscles, cytosolic PCr mostly serves as an energy store or as a cellular energy buffer (Fig. 3).

**Fig. 4:**
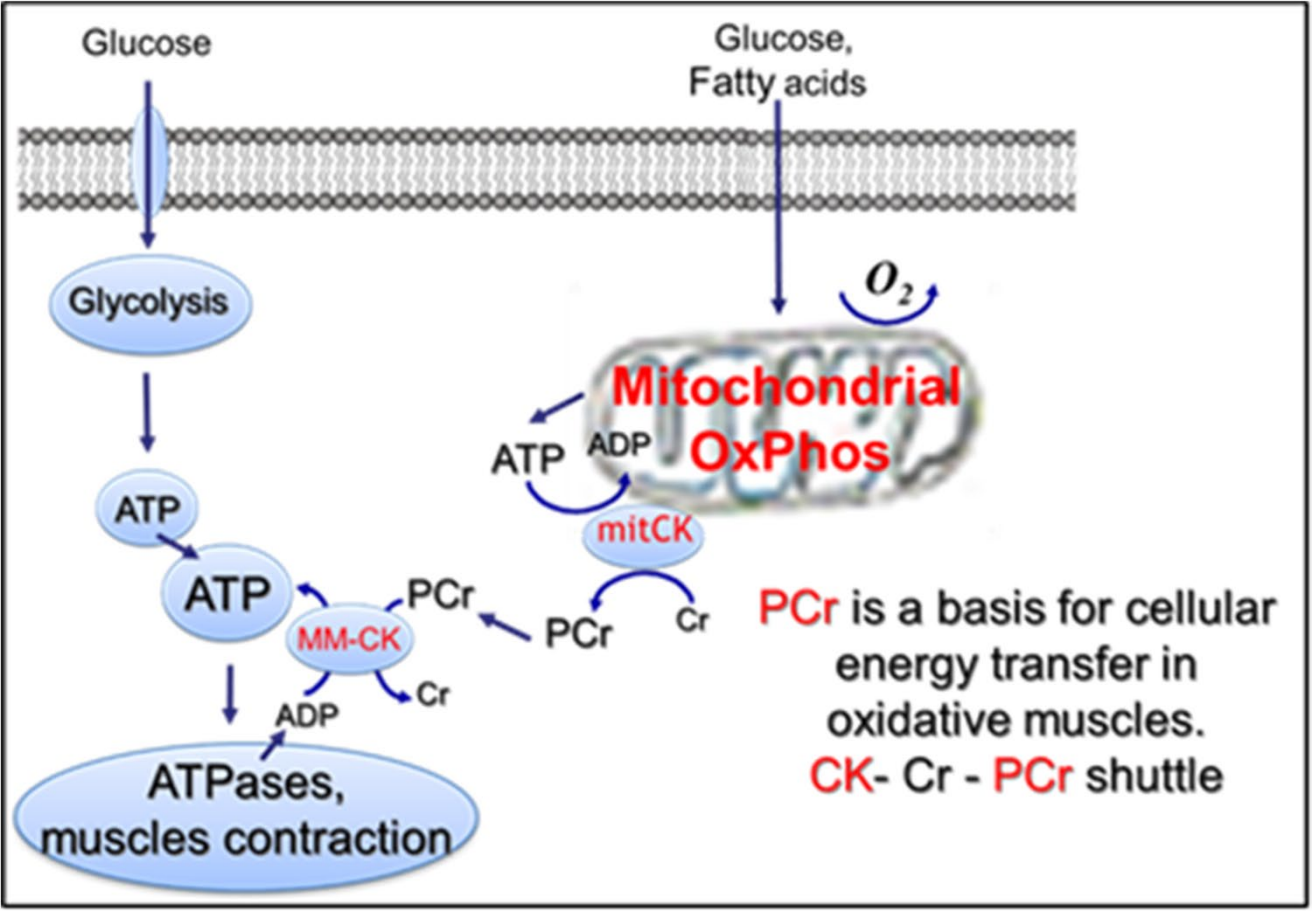
Energy metabolism mechanisms in the heart and oxidative, slow-twitch skeletal muscles

Mitochondrial respiratory function plays a central role in cellular energy metabolism and various redox regulations in the heart and oxidative skeletal muscles that are dependent on the aerobic ATP supply. Quite the opposite of glycolytic, in oxidative muscles, several isoforms of creatine kinase are involved in cell energy metabolism and intracellular energy transport (Fig. 4) for the synthesis of phosphocreatine in the mitochondrial intermembrane space, through the mitochondrial isoform of CK (mitCK). This mitCK (in the intermembrane space) is coupled to oxidative phosphorylation through the ATP-ADP translocator (ANT) in the inner mitochondrial membrane [23, 29, 52, 55]. Thus, the external addition of 30-mM creatine considerably increases mitochondrial respiration, simultaneously decreasing appKm for ADP (Table 1), due to mitCK-ANT coupling. In this case, mitCK operates as an ADP-regenerating system. In the same way, mitochondrial hexokinase may act as regenerating systems for ADP upon the addition of glucose. Previous studies showed that the coupling and functional state of mitCK could be significantly damaged in various muscle pathologies. A simple creatine test (creatine-stimulated respiration) can be used as a sensitive parameter and diagnostic tool in various injuries, such as oxidative stress, ischemia–reperfusion injury, and muscle dystrophies. In addition, numerous interactions between cytoskeletal proteins and mitochondria may participate in the regulation of mitochondrial respiratory function and OxPhos [23, 52]. In the heart and striated muscles, the positions of intermyofibrillar mitochondria are tightly fixed, providing their very regular arrangement. These may involve specific associations of cytoskeletal proteins with voltage-dependent anion channel (VDAC), thereby governing the permeability of the outer mitochondrial membrane (OMM) to various metabolites, regulating therefore mitochondrial respiration. Skeletal muscles, as well as muscle fibers, also demonstrate a regular arrangement of tubulin beta-II isoform entirely co-localized with mitochondria [23]. These studies suggest specific structural and functional coupling of this isoform of tubulin beta with mitochondria and participation of tubulin beta-II in the regulation of OMM permeability, which can also be regulated by the specific isoform of plectin [49, 63]. Specific isoforms of cytolinker protein plectin (plectin 1b and plectin 1 d) have been suggested as important cytoskeletal proteins in the regulation of mitochondrial function [63]. Therefore, previous studies strongly suggest the important role of the cytoskeleton in the regulation of energy metabolism.

Recent data show that tubulin beta II isoform and plectin 1b isoform can be involved in the control of fluxes via the energy-transferring super-complex VDAC-mitochondrial creatine kinase (ATP-ADP translocase), thus regulating the respiratory functions of mitochondria, bioenergetics, and therefore the entire cell physiology. Intermediate filament desmin represents an important component of the cytoskeleton. Desmin is an intermediate filament, and its damage can be involved in various disorders of skeletal muscles due to mutations in genes encoding desmin [39, 43]. These diseases may include desmin-related myopathies, desminopathy associated with dilated, restrictive, hypertrophic, and Emery–Dreifuss muscular dystrophy. In healthy muscles, desmin and other intermediate filament (IFs) proteins are responsible for the involvement of their network in mechano-signaling and mitochondrial physiology, whereas in diseases, their structure can be destroyed, leading to cell death and activation of inflammation [39].

## Possible supplementations for the improving of muscle performance after muscles injuries

Various interventions are applied and may be useful at improving both energy metabolism and skeletal muscle performance.

### Creatine

Creatine is a substance that is supplied to muscles via biosynthesis and diet. It is mostly localized in skeletal muscle and brain tissue, where it binds to phosphate to form phosphocreatine (PCr) via creatine kinase. From a bioenergetic point of view, creatine is essential in the energy metabolism of muscles under physiological and pathological conditions. It is a precursor of the energy metabolite–PCr, an important substance for the dynamic intracellular energy buffering in glycolytic muscles (Fig. 1) and intracellular energy transferring in the heart and oxidative skeletal muscles (Fig. 2). If its synthesis and cellular levels are impaired (e.g., in hereditary diseases), the lack of creatine may lead to malady muscle alterations. On the other hand, it has been shown that oral creatine supplementation can be effective in improving muscle function and muscular strength in various muscular injuries and dystrophies [4, 5, 24, 27]. Vegetarians and vegans who do not consume external creatine have a lower cellular concentration of creatine, and its supplementation, in this case, can be rather effective in improving muscular performance. Existing evidence demonstrates that creatine supplements can also be beneficial in muscular atrophy and sarcopenia during aging (see below). It has been shown that normal muscle function can also be improved in models of Duchenne muscular dystrophy (DMD) patients or mdx mice by restoring cellular creatine and energy metabolism. A short-term intake of creatine increases total skeletal muscle creatine content, as well as cellular PCr levels, demonstrating thus the benefits of creatine supplementation for muscular exercise performance due to elevated ATP production during exercise [24]. Some experimental evidence suggested that creatine supplementation could increase muscle strength and even mass during resistance training [27, 63]. Very recent data also show that creatine supplementation lightens eccentric exercise-induced muscle damage and inflammation [63]. Therefore, creatine is important and acts as an energy buffer in glycolytic (Fig. 1) and an energy transporter in oxidative muscles (Fig. 2, “The role of phosphocreatine for the efficient intracellular energy storage and transfer in different skeletal muscles” Section). It has also been suggested that creatine can diminish muscle damage from inflammation, having thus anti-inflammatory properties in peripheral tissues [63]. Accordingly, creatine continues to be one of the widely used sports-related, safe, and effective supplements, especially for athletes looking for explosive power.

### Melatonin

Melatonin is produced in organisms mostly at hours of darkness in humans. It has remarkable anti-inflammatory and antioxidant properties. The recent evidence shows the beneficial role of melatonin supplementation in skeletal muscle disorders due to its strong antioxidant effect [53]. In skeletal muscles, melatonin prevents muscle mitochondrial damage by reducing reactive oxygen species (ROS) overproduction, thus inhibiting oxidative stress and autophagy. Its beneficial role has been demonstrated in the recovery of skeletal muscle function and strength, maintaining the number of muscle fibers and reversing pathological changes of aging muscles (in patients with sarcopenia, see “Conclusion and future directions” section). Therefore, melatonin can be suggested as an effective dietary supplementation to prevent muscle injuries in aging-related sarcopenia disease [53]. Mitochondria are important targets of melatonin, protecting mitochondria and mitochondrial function by scavenging ROS, and therefore reducing cell oxidative stress [59]. In addition, many interactions between melatonin and miRNAs also suggest melatonin supplementation as a useful treatment of sarcopenia [28]. Melatonin is considered thus as a potent anti-sarcopenia and anti-aging agent, due to its multiple preventive effects, with positive treatment perspectives on muscle performance and physical activities [28].

### Caffeine

It has been shown that high doses of caffeine, just before exercise, may increase fatty acid oxidation and lipolysis during exercise, together with increased strength performance and decreased glycogen utilization in muscles. These effects appear to be due to increased catecholamine levels by caffeine [21, 22, 24].

### L-carnitine

L-carnitine provides a transport of fatty acids into mitochondria through the mitochondrial membrane, regulating the acetyl-CoA levels in the mitochondrial matrix and maintaining OxPhos and thus the entire mitochondrial bioenergetics [24, 60]. The muscle repair process is ATP demanding, and mitochondria provide an important source of energy for the period of muscle regeneration [60]. Mitochondrial fatty acids are shown to represent important energy substrates for muscle energy metabolism, particularly in oxidative muscles. During very high-intensity exercise; however, this process can be limited by the availability of L-carnitine to mitochondria. Therefore, supplementation with L-carnitine before exercise can be beneficial for muscle function and has been suggested to improve exercise performance. However, until now, there is no clear evidence that L-carnitine supplementation can really improve exercise performance at high exercise intensity. Experimental works show different results, depending on training status or duration of administration [19]. Moreover, supplementation with L-carnitine alone results in a minor increase in plasma and muscle L-carnitine content [24]. At the same time, it has been shown that the intake of L-carnitine together with large amounts of glucose may increase muscle L-carnitine levels. L-carnitine supplementation has also been suggested as useful in some diseases like liver cirrhosis and sarcopenia [17].

Moreover, some antioxidants and several amino acids may support muscle energy metabolism. Also, mitochondria-targeting agents can be considered as possible complementation against oxidative stress [2, 7, 47].

## Changes of skeletal muscle energy metabolism in pathology

Muscle repair is a high-energy demanding process, and mitochondrial OxPhos provides an important source for ATP during muscle regeneration. So, mitochondria are highly involved in the myogenesis process [10, 24, 29, 46, 55]. For normal muscle mass and energy demands, skeletal muscles should be active and able to consume various substrates. On the other hand, extended bed rest, muscle inactivity, and metabolic imbalance during trauma, illness, or various pathologies frequently may lead to skeletal muscle degeneration. Different mitochondrial sub-populations may be differently involved in both physiological and pathological processes, such as various skeletal muscle disorders. In addition, it has been shown that deprivation of substrates, such as glucose, serum, and growth factors may substantially increase subcellular mitochondrial heterogeneity in many aspects, including mitochondrial energization states (membrane potentials) in intact cells. The heterogeneous mitochondrial damage can be a result of different surrounding oxygen and Ca^2+^ concentrations or ROS levels, or due to heterogeneous cell redox states. Mitochondrial DNA has an increased number of mutations as compared with nuclear DNA. It has a lower ability to repair and less protection against ROS, and therefore may have more damage. Mitochondria and, in particular, mitCK are very sensitive to oxidative damage by ROS due to the oxidation of essential SH-residues. Alterations in micro-compartmentation of energy substrates (ATP, ADP, PCr) and in cellular energy transfer play a central role in the molecular, bioenergetic mechanisms of various muscle disorders. Therefore, supporting/protecting the mitCK system and the creatine-phosphocreatine energy transferring pathway may be considered important targets for possible protective interventions in muscle injuries.

### Mitochondrial myopathies

The genetic metabolic diseases are associated with DNA mutations, deletions, and severe mitochondrial dysfunction. They affect mostly skeletal muscles and are frequent inherited disorders in childhood [46]. Mitochondrial diseases can be involved also in other multiple organs, including peripheral nerves. Mitochondrial neuropathy and mitochondrial myopathy can be frequently linked at the level of the neuromuscular junction [38]. Mitochondrial biogenesis increases the respiration and OxPhos and many metabolic activities, whereas mitophagy serves to remove damaged or defective mitochondria from the cell. The important balance between these two opposing processes (biogenesis and mitophagy) is highly metabolically regulated [10].

### Duchenne’s muscular dystrophy

Duchenne muscular dystrophy (DMD) is another example of a genetic disease. It represents a severe, muscle-degenerative disorder [8, 16] and is characterized by a pronounced degradation of the structure of skeletal muscles, together with weakness of the plasma membrane, decreasing muscle strength. It has been shown that DMD is caused by the lack of functional dystrophin in the cell, due to dystrophin-encoding mutations. It has been found that mitochondria significantly deteriorate in this disease, leading to decreased OxPhos and hence impaired cellular bioenergetics. These changes are associated also with elevated cellular Ca^2+^ levels (Ca^2+^ homeostasis disruption) and alterations (weakness) of the cell plasma membrane [8, 41]. Glycolytic fibers are more vulnerable to DMD [6].

#### Animal model of Duchenne’s muscular dystrophy—mdx mice

The analysis of various muscles from mdx mouse, which is an animal model of the Duchenne muscular dystrophy (DMD), showed significant changes in mitochondrial function, associated with dystrophin deficiency. These studies have demonstrated that mitochondria from mdx mice skeletal muscles had nearly half of maximal respiration activities, measured by high-resolution respirometry of saponin-permeabilized muscle, as compared with controls [36]. Unfortunately, this study was limited to animal models; however, similar findings were observed in skeletal muscle biopsies from DMD patients. The experimental data demonstrated that the amount of several mitochondrial enzymes is decreased, possibly due to Ca^2+^ overload in muscles of mdx mice [36]. Interestingly, the heart mitochondria of mdx mice demonstrated no large changes, probably due to adaptation and upregulation of protein utrophin in their myocardium.

##### Effects of alcohol consumption on skeletal muscles

Chronic alcohol ill-use is associated with skeletal muscle weakness and denervation following muscle atrophy. There is strong evidence that problematic alcohol consumption can negatively affect skeletal muscle performance [9]. The negative effects of alcohol on skeletal muscle function and performance were obtained from clinical studies and using various animal models. The studies showed also that excessive alcohol consumption could negatively affect muscle recovery after intensive exercise. The mechanisms involved in muscle dysfunction may include the balance disruption between cellular anabolic and catabolic pathways, lower regeneration rate, increased inflammation, and mitochondrial function damage [9]. Experimental studies demonstrated that excessive alcohol consumption can significantly affect skeletal muscle performance through different mechanisms associated with worsened neuromuscular innervation and reduced skeletal muscle performance.

##### Eccentric exercise muscle damage

Eccentric exercise can be observed in various types of human physical activities. It has been demonstrated that eccentric exercise frequently may lead to significant muscle damage and muscle dysfunction [26]. This muscle damage may be associated with cell membrane diseases, degradation of myofibrillar proteins, inflammation, and mitochondrial dysfunction [26, 31].

## Aging and sarcopenia

Muscle weakness and its consequences compromise quality of life of older individuals and increase dependency on social and health care services [13, 15, 42, 62]. Sarcopenia has been referred to as a decline of skeletal muscle mass, strength, and functionality in old people [20, 30, 40]. It is associated with physical weakness, functional incapacity, tumbles, and hospitalizations and represents therefore one of the serious geriatric disorders. Sarcopenia can occur due to numerous factors, like reduced re-generative capacity, increased oxidative stress, dysfunction of mitochondria, and inflammation. The major cause of muscle weakness can be alterations in muscle cells bioenergetics due to reduced OxPhos in skeletal muscles [1, 12, 18, 37, 51, 58]. Mitochondria from aged muscles show less intensive ATP synthesis and less effective intracellular energy transfer that cannot provide adequate energy required for normal muscle performance. The muscle weakness in sarcopenia is associated with decreased muscle fiber cross-sectional area, impaired muscle quality, altered central and peripheral nervous system, changes in hormonal status, inflammation, lipotoxicity, and dysbalance between anabolic and catabolic effects [25, 37]. It has been hypothesized that muscle decline in sarcopenia might be caused by the direct detrimental effects of the chronic low-grade inflammatory status of progressive age. Aging and sarcopenia are associated with decreased metabolic rates due to reduced oxidative capacity of skeletal muscles. Oxidative damage (elevated reactive oxygen species, ROS generation) in sarcopenia is associated with many destructive processes (phospholipid peroxidation, DNA damage, etc.) [11, 14, 54, 56, 61]. It has been suggested that creatine and melatonin supplementations could be helpful at aging-dependent sarcopenia (see above). However, despite several efforts to study the effect of aging on mitochondria, molecular mechanisms and contribution of the altered mitochondrial activity, and dynamics to age-related muscle weakness continues to be debated. Presently, it is not known as a usable treatment to neutralize the loss of skeletal muscle mass and strength during aging.

## Conclusion and future directions

**Fig. 5:**
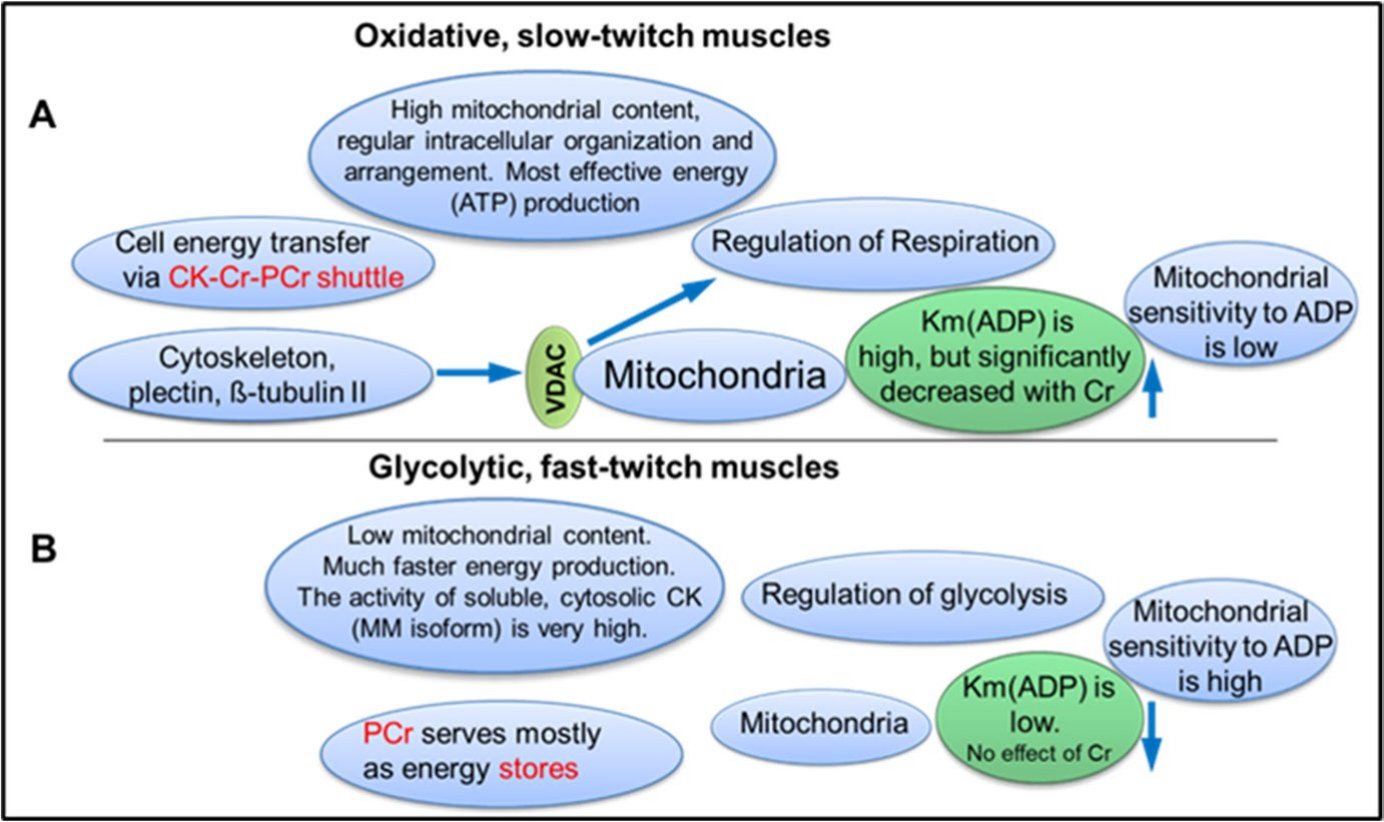
Bioenergetic features of oxidative (**A**) and glycolytic (**B**) muscles

Glycolytic and oxidative muscles demonstrate very different bioenergetic mechanisms (see Fig. 5).

Moreover, within the same muscle, both glycolytic and oxidative fibers can be present. Importantly, glycolysis and mitochondrial oxidative phosphorylation are in balanced complementation [50, 64]. One common feature of both pathways is the compartmentation of energy metabolites and their direct channeling. In oxidative muscles, the creatine kinase and phosphocreatine play an important role in their energy metabolism. In the glycolytic muscles, phosphocreatine, produced from creatine and ATP by cytosolic MMCK isoform, is considered an intracellular energy store necessary for fast ATP delivery, whereas in the oxidative skeletal muscles and in the myocardium, it is the main player in the intracellular energy transfer pathway. Mitochondrial importance has been demonstrated in various muscle diseases, such as various myopathies, age-related loss of muscle mass/function in sarcopenia, Duchenne muscular dystrophy, and so on.

Several interventions, which may modulate mitochondrial dynamics and turnover, can be useful to improve cellular bioenergetics and mitochondrial physiology after muscle injuries. Future studies are intended to further investigate and uncover molecular mechanisms of existing effective supplements and create new potent pharmacological agents that can target mitochondria, thus restoring muscle bioenergetics, performance, and muscle functional capacity [45].

## Data Availability

No datasets were generated or analysed during the current study.
